# Distribution of lymphoma subtypes in Ukraine according to the WHO 2016 classification

**DOI:** 10.1002/hon.3061

**Published:** 2022-08-19

**Authors:** Iryna Kriachok, Yana Stepanishyna, Tetiana Skrypets, Nazar Shokun, Arina Martynchyk, Iryna Tytorenko, Olena Aleksik, Mykhailo Krotevych, Martina Manni, Massimo Federico

**Affiliations:** ^1^ Department of Oncohematology National Cancer Institute Kyiv Ukraine; ^2^ Department of Bone Marrow Transplant National Cancer Institute Kyiv Ukraine; ^3^ Surgical, Medical and Dental Department of Morphological Sciences Related to Transplant Oncology and Regenerative Medicine University of Modena and Reggio Emilia Modena Italy; ^4^ PhD School in Clinical and Experimental Medicine University of Modena and Reggio Emilia Modena Italy; ^5^ Pathology Department National Cancer Institute Kyiv Ukraine

**Keywords:** malignant lymphomas, lymphoma registry, WHO lymphoma classification

## Abstract

The Ukrainian Lymphoma Registry (ULR) was established in 2019 with the aim of monitoring the quality of diagnosis, staging, and treatment of lymphoma in Ukraine. Between September 2019 and October 2021, 546 patients with newly diagnosed lymphoma were prospectively registered. All cases were diagnosed according to the 2016 updated WHO lymphoma classification. The male‐to‐female ratio (M/F) for the whole population was 0.7, with a median age of 46 years (range 18–95). The adoption of the 2016 WHO classification resulted in the identification of 36 different lymphoma subtypes, with 132 cases (24.2%) classified differently compared to the 2008 WHO classification. Only 12 cases (2.8%) were true new entities, including seven cases of high‐grade B‐cell lymphoma NOS, three of anaplastic large B‐cell lymphoma, ALK‐negative, 1 case of HHV8+ DLBCL NOS, and 1 of high‐grade B‐cell lymphoma with C‐MYC and BCL2/BCL6 rearrangement. Moreover, 55 (61.1%) entities, including 37 defined by WHO 2008 and 18 defined by WHO 2016, were not represented at all. The analysis of cases registered in the ULR provides a comprehensive breakdown of the subtypes, stage distribution, and treatment of malignant lymphomas (ML) in Ukraine, supporting the usefulness of prospective data collection and timely reporting. We believe that this study is the first step toward a better understanding of the real‐life outcomes of patients with ML.

## PEER REVIEW

The peer review history for this article is available at https://publons.com/publon/10.1002/hon.3061.

## 1 INTRODUCTION

Malignant Lymphomas (ML) are a heterogeneous group of disease accounting to more than 100 different subtypes in the last WHO classification of hematopoietic and lymphoid tumors published in 2016.^1^ According to the Ukrainian National Cancer Registry (UNCR), 3211 patients have been diagnosed with ML in 2019 (875 cases of Hodgkin Lymphoma (HL) and 2336 Non‐Hodgkin Lymphoma (NHL)).^2^

Despite the UNCR is quite accurate, it lacks information on histological sub‐types and patient characteristics, treatment details and outcome. To overcome these limitations, in 2019 the Ukrainian Lymphoma Registry (ULR) was established.

## 2 METHODS

The ULR prospectively collected information on clinical and disease characteristics, first‐line treatment, and response of all ML cases classified according to the WHO 2016 classification.^1^ Cases initially diagnosed outside the Pathology Department of National Cancer Institute of Kyiv were revised by two national expert hemopathologists. Registration was performed on a secured dedicated database. The study was approved by the Institutional Review Board of the National Cancer Institute of Kyiv.

## 3 RESULTS

Between September 2019 and October 2021, 546 patients with newly diagnosed ML were registered, including 348 (63.7%) cases of B‐NHL, 18 cases (3.2%) of Peripheral, non‐cutaneous, T‐Cell Lymphomas (PTCL), and 180 cases (33%) of HL.

The male to female ratio (M/F) for the whole population was 0.7, and the overall median age was 45.8 years (18–95). 447 (81.9%) patients had less than 65 years, and 99 (18.1%) were over 65. Two hundred and seventy‐six cases (50.5%) had pure nodal disease, and EN involvement was observed in 275 cases (50.3%) with 90 cases reclassified as Primary Extranodal lymphoma (16.5%). Advanced stage was diagnosed in 85 cases (47%) of HL, 217 (62.4%) of B‐NHL and in 10 (55%) of T‐NHL.

Overall, the adoption of the WHO 2016 classification resulted in the recognition of 36 different lymphoma subtypes, with 132 cases (24.2%) classified differently than the WHO 2008 classification. Of note, only 12 cases (2.8%) were true new entities: 7 cases of high‐grade B‐cell lymphoma NOS, 3 of Anaplastic large B cell lymphoma, ALK‐, 1 case of HHV8+ DLBCL NOS, and 1 of high‐grade B‐cell lymphoma with C‐MYC and BCL2/BCL6 rearrangement.

**FIGURE 1:**
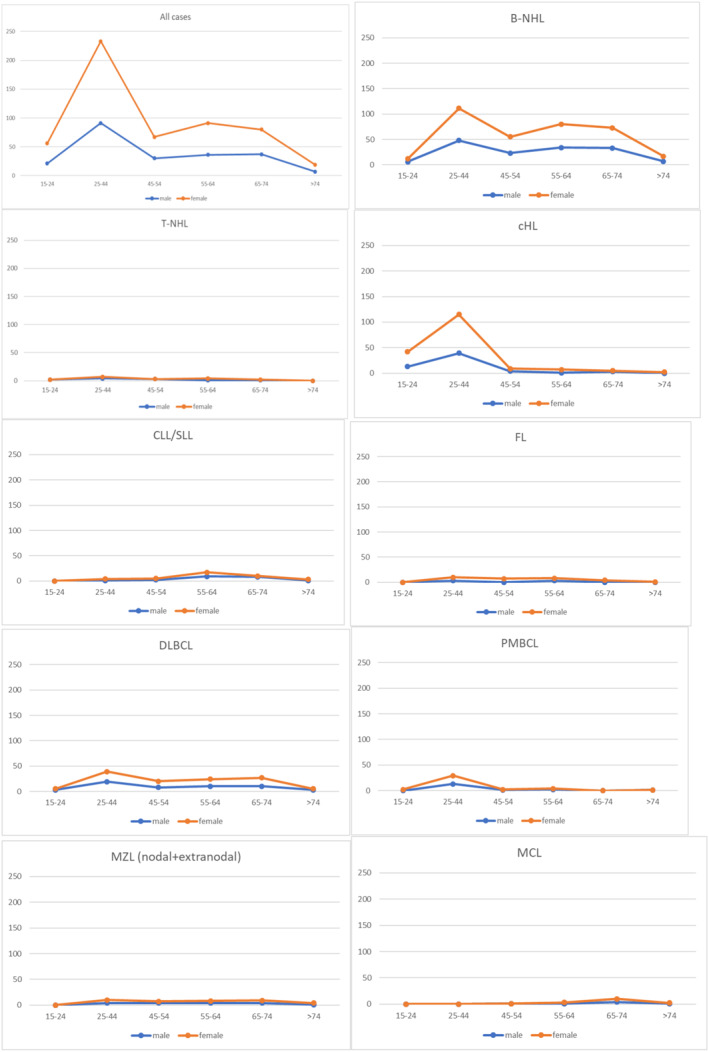
Incidence of lymphoid neoplasms by subtype, age and sex. B‐cell non‐Hodgkin lymphoma (B‐NHL), T‐cell non‐Hodgkin lymphoma (T‐NHL) classical Hodgkin lymphoma (cHL), Chronic lymphocytic leukemia/small lymphocytic lymphoma (CLL/SLL), follicular lymphoma (FL), diffuse large B‐cell lymphoma (DLBCL), primary mediastinal B‐cell lymphoma (PMBCL), marginal zone lymphoma (MZL), mantle cell lymphoma (MCL)

Interestingly, up to 55 different subtypes were not recorded in ULR, including 37 entities already defined by the WHO 2008, and 18 new entities defined by WHO 2016 (Table 1). Figure 1 shows the incidence of lymphoid neoplasms by subtype, age, and sex. Treatment details are summarized in Table 2.

**TABLE 1:** Frequency of the analyzed lymphoma subtypes

	Subtypes	N (%)	M:F	≤18 years	19‐64 years	>65 years	Median age (years)
Hodgkin lymphoma		180 (33)					30.3
	Nodular lymphocyte predominant	5 (2.8)	4:1	0	5	0	32.4
	cHL	175 (97.2)					
	Nodular sclerosis	124 (68.9)	40:84	10	110	4	30.3
	Lymphocyte‐rich	7 (3.9)	2:5	1	6	0	37.7
	Mixed cellularity	29 (16.1)	10:19	0	28	1	31.7
	Lymphocyte‐depleted	6 (3.3)	1:5	0	5	1	44.5
	Unspecified	9 (5)	3:6	0	8	1	36.2
B‐cell neoplasms		348 (63.7)					51.7
	B‐CLL/SLL	39 (11.2)	21:18	0	26	13	60
	Splenic MZL	6 (1.7)	2:4	0	6	0	45.4
	Waldenstrom macroglobulinemia	3 (0.9)	2:1	0	2	1	55.6
	Extranodal MZL (MALT lymphoma)	21 (6)	10:11	0	14	7	55.5
	Nodal MZL	17 (4.9)	7:10	0	11	6	60.1
	Follicular lymphoma	29 (8.3)	6:23	0	24	5	53.3
	Mantle cell lymphoma	17 (4.9)	8:9	0	4	13	64.8
	Diffuse large B cell lymphoma	120 (34.5)					
	DLBCL (GCB)	**44 (12.6)**	**20:24**	**0**	**33**	**11**	**56.5**
	DLBCL (ABC)	**63 (18.1)**	**27:36**	**0**	**44**	**19**	**50.2**
	DLBCL NOS	**13 (3.7)**	**6:7**	**1**	**10**	**2**	**37.2**
	T cell/histiocyte‐rich large B‐cell lymphoma	2 (0.6)	0:2	0	2	0	49.2
	Primary central nervous system lymphoma	29 (8.3)	13:16	0	19	9	55.5
	Primary cutaneous DLBCL, leg type	4 (1.1)	1:3	0	2	2	65.7
	Primary mediastinal large B‐cell lymphoma	38 (10.9)	17:21	0	37	1	38.9
	ALK‐positive large B‐cell lymphoma	2 (0.6)	2:0	0	2	0	40.1
	HHV8‐positive DLBCL, NOS	**1 (0.3)**	**1:0**	**0**	**1**	**0**	**48**
	Burkitt lymphoma	5 (1.4)	2:3	1	4	0	34.1
	BCLU intermediate between DLBCL and cHL	5 (1.4)	2:3	0	5	0	33.2
	High grade B‐cell lymphoma with MYC and BCL2 and/or BCL6 rearrangements	**1 (0.3)**	**1:0**	**0**	**1**	**0**	**63**
	HG B‐cell lymphoma, NOS	**7 (2)**	**3:4**	**0**	**6**	**1**	**54.4**
	Lymphoma/leukemia, unclassifiable	1 (0.3)	0:1	0	1	0	63
	Lymphoplasmacytic lymphoma	1 (0.3)	0:1	0	1	0	41
NK/T‐cell neoplasms		18 (3.2)					43.7
	T‐cell large granular lymphocytic leukemia	3 (16.7)	2:1	1	2	0	33.5
	Adult T‐cell leukemia/lymphoma	1 (5.5)	0:1	0	1	0	40
	Enteropathy‐associated T‐cell lymphoma	1 (5.5)	1:0	0	1	0	44
	Peripheral T‐cell lymphoma, NOS	3 (16.7)	3:0	0	3	0	42.3
	Angioimmunoblastic T‐cell lymphoma	3 (16.7	1:2	0	1	2	56.7
	Anaplastic large cell lymphoma, ALK‐positive	3 (16.7)	2:1	0	3	0	43.8
	Anaplastic large cell lymphoma, ALK‐negative	**3 (16.7)**	**1:2**	**0**	**3**	**0**	**37.2**
	Subcutaneous panniculitis‐like T‐cell lymphoma	1 (5.5)	1:0	0	1	0	52
Total		546					

*Note*: Bold value, new entities defined by WHO 2016.

Abbreviations: ABC, activated B‐cell; ALK, anaplastic lymphoma kinase; B‐CLL/SLL, Chronic lymphocytic leukemia/small lymphocytic lymphoma; BCLU, B‐cell lymphoma unclassifiable; cHL, classical Hodgkin lymphoma; DLBCL, diffuse large B‐cell lymphoma; GCB, germinal center B‐cell; MALT, Mucosa‐associated lymphoid tissue; MZL, marginal zone lymphoma; NOS, not otherwise specified.

**TABLE 2:** “Initial therapy” of the most frequent subtypes

	N, % completed from all registered	ABVD	BEACOPP escalated	R‐ABVD (for LP)			
cHL	119 (66.1%)	113 (95%)	3 (2.5%)	3 (2.5%)			

		**RB**	**R‐FC**	**Other**			
B‐CLL/SLL	30 (76.9%)	28 (93.3%)	1 (3.33%)	1 (3.33%)			

		**RB**	**R‐CVP**	**R‐monotherapy**	**R‐CHOP**	**R‐CVP**	**Other**
MZL nodal	13 (76.5%)	12 (92.3%)	1 (7.7%)				
MZL (MALT)	14 (66.7%)	11 (78.6%)	‐	1 (7.1%)	1 (7.1%)		
FL	26 (70.3%)	9 (34.6%)		6 (23.1%)	10 (38.5%)	1	

		**RB**	**R‐CHOP/R‐DHAP**	**Other**			
MCL	12 (70.6%)	10 (83.3%)	1 (7.7%)	1 (7.7%)			

		**R‐CHOP**	**Dose‐adjusted EPOCH‐R**	**R‐mini‐CHOP**	**Beginning from cytoreduction (Cycl + Prednisone) then R‐CHOP**	**1R‐ACOD9 + 6R‐CHOP**	**Other**
DLBCL (all)	104 (92.9%)	63 (60.6%)	5 (4.8%)	10 (9.6%)	13 (12.5%)	9 (8.7%)	4 (3.8%)
PMLCL	27 (71.1%)	4 (14.8%)	21 (77.8%)	‐	‐	‐	2 (7.4%)

		**R‐MPV**	**AraC consolidation**	**HDCT + ASCT**			
PCNSL	20 (69%)	20 (100%)	5	4			

Abbreviations: ABVD, doxorubicin, bleomycin, vinblastine and dacarbazine; AraC, Cytarabine; ASCT, autologous stem cell transplant; B‐CLL/SLL, Chronic lymphocytic leukemia/small lymphocytic lymphoma; BEACOPP, Bleomycin, Etoposide, Doxorubicin, Cyclophosphamide, Vincristine, Procarbazine, Prednisolone; cHL, classical Hodgkin lymphoma; DLBCL, diffuse large B‐cell lymphoma; EPOCH‐R, etoposide, prednisone, vincristine, cyclophosphamide, doxorubicin, rituximab; FL, Follicular lymphoma; HDCT, high dose chemotherapy; MALT, Mucosa‐associated lymphoid tissue; MCL, mantle cell lymphoma; MZL, marginal zone lymphoma; PCNSL, Primary central nervous system lymphoma; PMBCL, Primary mediastinal large B‐cell lymphoma; R, rituximab; R‐ACOD, rituximab, adriamycin, cyclophosphamide, vincristine, prednisone; RB, rituximab and bendamustine; R‐CHOP, rituximab, cyclophosphamide, doxorubicin hydrochloride, vincristine, prednisolone; R‐CVP, rituximab, cyclophosphamide, vincristine, prednisolone; R‐DHAP, rituximab, dexamethasone, cytarabine, cisplatin; R‐FC, rituximab, fludarabine, cyclophosphamide; R‐MPV, rituximab, methotrexate, procarbazine, vincristine.

## 4 DISCUSSION

An important element that pervades many parts of the 2016 WHO lymphoma classification derives from new clinical, pathological, and genetic/molecular data that allow the recognition of different entities. However, it seems that many new data apply to conditions that are not frequent in daily practice. In fact, although 36 different lymphoma subtypes were recorded by the ULR, 55 different entities, 37 defined by WHO 2008 and 18 by WHO 2016, are not represented in the database with at least one case, yet. When evaluating our data on the frequency of B‐NHL, they compare favorably with those reported in several western countries.^3^, ^4^ Moreover, the proportion of cases of HL (33%) was higher than in other lymphoma registries,^5^, ^6^ reflecting the younger median age of Ukrainian population and probably the role of referral center exerted by UNCI.

Of note, there were differences in the frequencies of some histological subtypes between those recorded by the URL and published data. With 10.9% and 8% of B‐NHL, PMBCL and PCNSL were more frequent compared to other data sources.^7^, ^8^, ^9^ In literature, the incidence of PMBCL and PCNSL was by far below, being approximately 2%–4% and 1%–2% of NHLs, respectively. The high number of cases identified by the ULR could be explained by its role of tertiary care center offering up to date treatment to patients with these rare disorders. Cases of CLL/SLL accounted for 11% and cases of FL to 8.3% of B‐NHL. These figures are lower than those reported in western countries, where they account to 20%–30% for CLL and 12%–20% for FL.^3^ At the same time in the recently published trends by Swedish lymphoma registry, the rate of FL is 16%.^7^ This difference could be explained by the younger study population, the reduced rate of hospital admissions and the exclusion of patients who do not need active therapy.

Interestingly, few cases of mature T‐NHL have been found, and no cases of Follicular helper T‐cell related lymphomas (TFH) were recorded. There is no clear explanation for this observation, mostly because TFH related lymphomas are one of the new forms of PTCLs and the diagnosis is could be misprinted as a reactive follicular hyperplasia.^8^

Although the 2016 WHO classification of HLs has not changed compared to the 2008 edition, the former provides some updates concerning nodular lymphocyte–predominant HL (NLPHL). In the ULR registry only 5 out of 180 HL case (2.8%) have been recorded as NLPHL. Currently, due to the small number of NLPHL cases, it is difficult to assess any comparison with cases of classic HL.

An additional aim of the present study was the assessment of the quality of care provided to the patients. Bendamustine based regimens were used in indolent lymphomas and DA‐EPOCH‐R mostly used in PMBCL, as shown in Table 2. Of note, in HL the preference goes for ABVD as standard approach and the use of ABVD plus Rituximab in patients with NLPHL. The ULR suggests that the adoption of the WHO 2016 had a very limited impact on treatment decisions, although the new classification may have had a relevant impact on a better understanding of the biology of different lymphomas.

In conclusion, our preliminary ULR data provides a comprehensive analysis on the distribution of ML in Ukraine, classified according to the current 2016 WHO classification. As expected, considering the Ukrainian population age structure, a high percentage of ML are represented by HL, while a limited number of FL, CLL/SLL and T‐NHL have been found. Regardless its very young age, ULR may help to better investigate the outcome of patients with ML outside clinical trials, in the real world.

## CONFLICT OF INTEREST

All authors declare no competing financial interests.

## Supporting information

Supporting Information S1

## Data Availability

The data that support the findings of this study are available from the corresponding author upon reasonable request.
