# Supplementary material for: Distribution of lymphoma subtypes in Ukraine according to the WHO 2016 classification
Source: Hematol Oncol. 2022 Aug 19;41(1):196–200. doi: 10.1002/hon.3061 (PMC10087250; doi:10.1002/hon.3061)
Supplement: Supplementary file 1 — Supporting Information S1 [file HON-41-196-s001.docx]

**SUPPLEMENTARY MATERIAL.**

**Supplementary Table 1.** Clinical features of Hodgkin lymphoma cases. *

|  | **NLP** | **cHL** | | | | |
| --- | --- | --- | --- | --- | --- | --- |
|  |  | **NS** | **MC** | **LD** | **LR** | **Still uncl** |
| **number** | 5 | 124 | 29 | 6 | 7 | 9 |
| **Median age (Range)** | 32.4  (26-34) | 30.3  (18-77) | 31.7  (19-76) | 44.5  (27-73) | 37.7  (18-61) | 36.2  (21-69) |
| **PS** |  |  |  |  |  |  |
| **0-1** | 5 (100) | 117 (94.4) | 29(100) | 5 (83.3) | 6 (85.7) | 8 (88.9) |
| **2-4** | 0 | 7 (5.6) | 0 | 1 (16.7) | 1 (14.3) | 1 (11.1) |
| **NA** | - | - | - | - |  | - |
|  |  |  |  |  |  |  |
| **AA stage** |  |  |  |  |  |  |
| **I** | 2 (40) | 3 (2.4) | 0 | 0 | 0 | 0 |
| **II** | 0 | 61 (49.2) | 18(62.1) | 3 (50) | 2 (28.6) | 6 (66.7) |
| **III** | 3 (60) | 28 (22.6) | 4 (13.8) | 1 (16.7) | 4 (57.1) | 1 (11.1) |
| **IV** | 0 | 32 (25.8) | 7 (24.1) | 2 (33.3) | 1 (14.3) | 2 (22.2) |
| **NA** |  |  |  |  |  | - |
|  |  |  |  |  |  |  |
| **BM** |  |  |  |  |  |  |
| **Positive** | 0 | 3 (4.1) | 2 (9.1) | 0 | 0 | 0 |
| **Negative** | 3 (100) | 70 (95.9) | 20 (90.9) | 4 (100) | 6 (100) | 8 (88.9) |
| **NA** | 2(40) | 51 (41.1) | 7 (24.1) | 2 (33.3) | 1 (14.3) | 1 (11.1) |
|  |  |  |  |  |  |  |
| **ENS** |  |  |  |  |  |  |
| **0** | 4 (80) | 60 (51.3) | 14 (50.0) | 3 (50) | 4 (57.1) | 4 (50) |
| **1** | 0 | 31 (26.5) | 6 (21.4) | 1 (16.7) | 1 (14.3) | 4 (50) |
| **2+** | 1(20) | 26 (22.2) | 8 (28.6) | 2 (33.3) | 2 (28.6) | 0 |
| **NA** | - | 7 (5.6) | - |  | - | 1 (11.1) |
|  |  |  |  |  |  |  |
| **B-symptoms** | 2 (40) | 51 (41.1) | 14 (48.3) | 5 (83.3) | 5 (71.4) | 4 (44.4) |
|  |  |  |  |  |  |  |
| **LDH>UNL** | 0 | 13 (10.5) | 5 (17.2) | 0 | 0 | 3 (33.3) |
| **NA** | 1 (20) | 9.7 | 3.4 | 16.7 | 0 | 11.1 |

Abbreviations. NLP: Nodular lymphocyte predominant; NS: nodular sclerosis; MC: mixed cellularity; LD: lymphocyte-depleted; LR: lymphocyte-rich; PS: Performance status; NA: not assessed; AA: Ann Arbor; BM: bone marrow; ENS: extranodal sites; LDH: lactate dehydrogenase; UNL: upper normal limit.

**Supplementary Table 2.** Clinical features of B-cell non-Hodgkin lymphoma cases. *

|  | **CLL/**  **SLL** | **splenic MZL** | **MALT** | **nodal MZL** | **FL** | **MCL** | **DLBCL**  **GCB** | **DLBCL**  **ABC** | **DLBCL**  **NOS** | **PCNSL** | **PMBCL** | **BL** | **LPL** |
| --- | --- | --- | --- | --- | --- | --- | --- | --- | --- | --- | --- | --- | --- |
| **number** | 39 | 6 | 21 | 17 | 29 | 17 | 44 | 63 | 13 | 29 | 38 | 5 | 1 |
| **Median age (Range)** | 60  (33-79) | 45.4  (33-60) | 55.5  (30-78) | 60.1  (28-83) | 53.3  (29-79) | 64.8  (52-84) | 56.5  (22-79) | 50.2  (21-100) | 37.2  (17-71) | 55.5  (21-75) | 38.9  (19-77) | 34.1  (17-60) | 41 |
| **PS** |  |  |  |  |  |  |  |  |  |  |  |  |  |
| **0-1** | 31(79.5) | 6 (100) | 20 (95.2) | 17 (100) | 27 (93.1) | 14 (82.4) | 31 (70.5) | 52 (82.5) | 11 (84.6) | 14 (48.3) | 27 (71) | 3 (60) | 1 (100) |
| **2-4** | 8 (20.5) | 0 (0) | 1 (4.8) | 0 (0) | 2 (6.9) | 3 (17.6) | 13 (29.5) | 11 (17.5) | 2 (15.4) | 15 (51.7) | 10 (29) | 2 (40) | 0 (0) |
| **NA** |  |  |  | - |  | - |  |  |  |  | 1 (2.6) |  |  |
|  |  |  |  |  |  |  |  |  |  |  |  |  |  |
| **AA stage** |  |  |  |  |  |  |  |  |  |  |  |  |  |
| **I** | 1 (2.6) | 0 (0) | 3 (14.3) | 1 (5.9) | 1 (3.4) | - | 7 (15.9) | 13 (20.6) | 1 (7.7) | - | 10 (26.3) | 0 | 0 |
| **II** | 8 (20.5) | 1 (16.7) | 1 (4.8) | 3 (17.6) | 4 (13.8) | - | 12 (27.3) | 15 (23.8) | 5 (38.5) | - | 15 (39.5) | 1 (20) | 1 (100) |
| **III** | 9 (23) | 0 (0) | 2 (9.5) | 4 (23.5) | 17 (58.6) | 1 (5.9) | 9 (20.5) | 7 (11.1) | 2 (15.4) | - | 2 (5.3) | 2 (40) | 0 |
| **IV** | 21(53.8) | 5 (83.3) | 15 (71.4) | 9 (52.9) | 7 (24.1) | 16 (94.1) | 16 (36.4) | 28 (44.4) | 5 (38.5) | - | 11 (28.9) | 2 (40) | 0 |
| **NA** |  |  |  |  |  |  | - | - |  |  |  |  |  |
|  |  |  |  |  |  |  |  |  |  |  |  |  |  |
| **BM** |  |  |  |  |  |  |  |  |  |  |  |  |  |
| **Positive** | 28 (90.3) | 4 (66.7) | 5 (35.7) | 7 (46.7) | 5 (21.7) | 7 (63.6) | 4 (11.8) | 4 (9.3) | 0 | 1 (6.25) | 0 | 2 (40) | 0 |
| **Negative** | 3 (9.7) | 2 (33.3) | 9 (64.3) | 8 (53.3) | 18 (78.3) | 4 (36.4) | 30 (88.2) | 39 (90.7) | 4 (100.0) | 15 (93.7) | 23 (100?) | 1 | 1 |
| **NA** | 8 (20.5) | - | 7 (33.3) | 2 (11.8) | 6 (20.7) | 6 (35.3) | 10 (22.7) | 20 (31.7) | 9 (69.2) | 13 (44.8) | 15 (39) | 2 (40) | - |
|  |  |  |  |  |  |  |  |  |  |  |  |  |  |
| **ENS** |  |  |  |  |  |  |  |  |  |  |  |  |  |
| **0** | 33 (84.6) | 6 (100) | - | 7 (41.2) | 10 (35.7) | 6 (35.3) | 13 (30.2) | 22 (35.5) | 1 (7.7) | - | 18 (47.3) | 2 (40) | 1 |
| **1** | 3 (7.7) | 0 | - | 5 (29.4) | 11 (39.3) | 4 (23.5) | 15 (34.9) | 9 (14.5) | 6 (46.2) | - | 15 (39.5) | 1 (20) | 0 |
| **2+** | 3 (7.7) | 0 | 8 (38) | 5 (29.4) | 7 (25.0) | 7 (41.2) | 15 (34.9) | 31 (50) | 6 (46.2) | - | 5 (13.2) | 2 (40) | 0 |
| **NA** |  |  |  |  | 1 (3.4) |  | 1(2.3) | 1 (1.6) |  |  |  |  |  |
|  |  |  |  |  |  |  |  |  |  |  |  |  |  |
| **B-symptoms** | 18 (46.1) | 2 (33.3) | 3 (14.3) | 5 (29.4) | 7 (24.1) | 7 (41.2) | 20 (45.5) | 24 (38.1) | 7 (53.8) | 3 (10.3) | 16  (42.1) | 3 (60) | 0 |
|  |  |  |  |  |  |  |  |  |  |  |  |  |  |
| **LDH>UNL** | 5 (12.8) | 1 | 1 | 4 (23.5) | 2 (6.9) | 5 (29.4) | 16 (36.4) | 16 (28.6) | 4 (30.8) | 6 (20.6) | 15  (39.5) | 4 (80) | 1 |
| **NA** | 12.5 | 0 | 4.7 | 11.4 | 2 (6.9) | 23.5 | 3 (6.8) | 7 (11.1) | 2 (15.4) | 6.8 | 0 | 20 | 0 |

Abbreviations. CLL/SLL: Chronic lymphocytic leukemia/small lymphocytic lymphoma; MZL: marginal zone lymphoma; MALT: Mucosa-associated lymphoid tissue; FL: follicular lymphoma; MCL: mantle cell lymphoma; DLBCL: diffuse large B-cell lymphoma; GCB: germinal center B-cell; ABC: activated B-cell; NOS: not otherwise specified; PCNSL: primary central nervous system lymphoma; PMBCL: Primary mediastinal large B-cell lymphoma; BL: Burkitt lymphoma; LPL: lymphoplasmacytic lymphoma; PS: Performance status; NA: not assessed; AA: Ann Arbor; BM: bone marrow; ENS: extranodal sites; LDH: lactate dehydrogenase; UNL: upper normal limit.

**Supplementary Table 3.** Clinical features of T-cell non-Hodgkin lymphoma cases. *

|  | **TLGL** | **ATLL** | **EATL** | **SPTCL** | **PTCL, NOS** | **ALCL, ALK+** | **ALCL, ALK-** | **AITL** |
| --- | --- | --- | --- | --- | --- | --- | --- | --- |
| **number** | 3 | 1 | 1 | 1 | 3 | 3 | 3 | 3 |
| **Median age (Range)** | 33.5  (18-52) | 40  (-) | 44  (-) | 52  (-) | 42.3  (35-47) | 43.8  (34-57) | 37.2  (20-55) | 56.7  (37-67) |
| **PS** |  |  |  |  |  |  |  |  |
| **0-1** | 2 (66.7) | 1 (100) | 1 (100) | 1 (100) | 2 (66.7) | 3 (100) | 3 (100) | 3 (100) |
| **2-4** | 1 (33.3) | - | - | - | 1 (33.3) | 0 | 0 | 0 |
| **NA** |  |  |  |  |  |  |  |  |
|  |  |  |  |  |  |  |  |  |
| **AA stage** |  |  |  |  |  |  |  |  |
| **I** | 0 | - | - | - | 1 (33.3) | 1 (33.3) | 2 (66.7) | 1 (33.3) |
| **II** | 1 (33.3) | - | - | 1 (100) | 0 | 1 (33.3) | 0 | 0 |
| **III** | 1 (33.3) | 1 (100) | - | - | 2 (66.7) | 1 (33.3) | 0 | 1 (33.3) |
| **IV** | 1 (33.3) | - | 1 (100) | - | 0 | 0 | 1 (33.3) | 1 (33.3) |
| **NA** |  |  |  |  |  |  |  |  |
|  |  |  |  |  |  |  |  |  |
| **BM** |  |  |  |  |  |  |  |  |
| **Positive** | 1 (33.3) | 0 | 1 (100) | 0 | 1(33.3) | 0 | 1 (33.3) | 2 (66.7) |
| **Negative** | 2 (66.7) | 1 (100) | 0 | 1 (100) | 2 (66.7) | 3 (100) | 1 (33.3) | 0 |
| **NA** | - | - | - | - | - | - | 1 (33.3) | 1 (33.3) |
|  |  |  |  |  |  |  |  |  |
| **ENS** |  |  |  |  |  |  |  |  |
| **0** | 2 (66.7) | 1 (100) |  | 0 | 2 (66.7) | 2 (66.7) | 2 (66.7) | 3 (100) |
| **1** | - | 0 |  | 1 (100) | 1 (33.3) | 0 | 1 (33.3) | 0 |
| **2+** | 1 (33.3) | 0 |  | 0 | 0 | 1 (33.3) | 0 | 0 |
| **NA** |  |  |  |  |  |  |  |  |
|  |  |  |  |  |  |  |  |  |
| **B-symptoms** | 1 (33.3) | 0 | 0 | 0 | 2 (66.7) | 1 (33.3) | 2 (66.7) | 0 |
|  |  |  |  |  |  |  |  |  |
| **LDH>UNL** | 0 | 1 (100) | 1 (100) | 0 | 1 (33.3) | 0 | 1 (33.3) | 2 (66.7) |
| **NA** | 1(33.3) | - | - | - | - | - | - | - |

Abbreviations. TLGL: T-cell large granular lymphocytic leukemia; ATLL: Adult T-cell leukemia/lymphoma; EATL: Enteropathy-associated T-cell lymphoma; SPCTL: Subcutaneous panniculitis-like T-cell lymphoma; PTCL,NOS: Peripheral T-cell lymphoma, not otherwise specified; ALCL: Anaplastic large cell lymphoma; ALK: anaplastic lymphoma kinase; AITL: angioimmunoblastic T-cell lymphoma; PS: Performance status; NA: not assessed; AA: Ann Arbor; BM: bone marrow; ENS: extranodal sites; LDH: lactate dehydrogenase; UNL: upper normal limit.

Figure 1. Incidence of lymphoid neoplasms by subtype, age and sex.

**
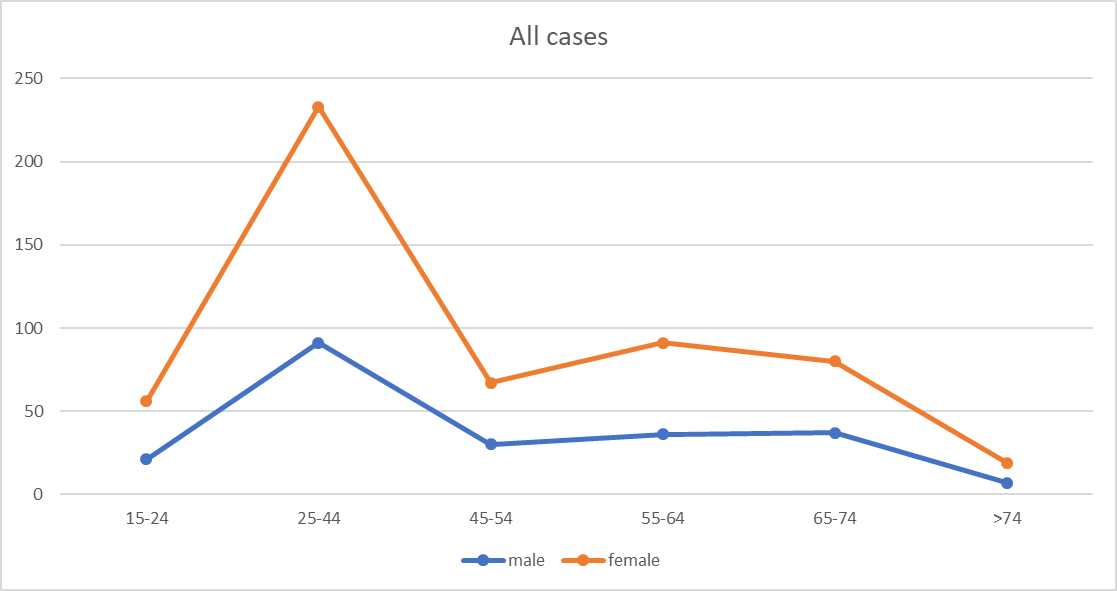

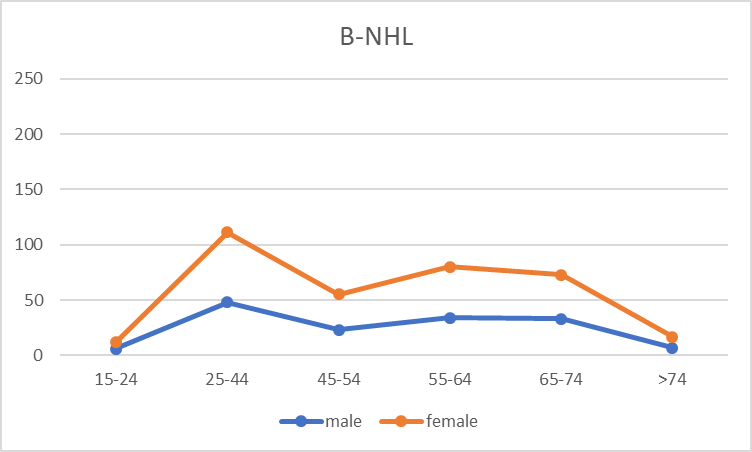
**

**
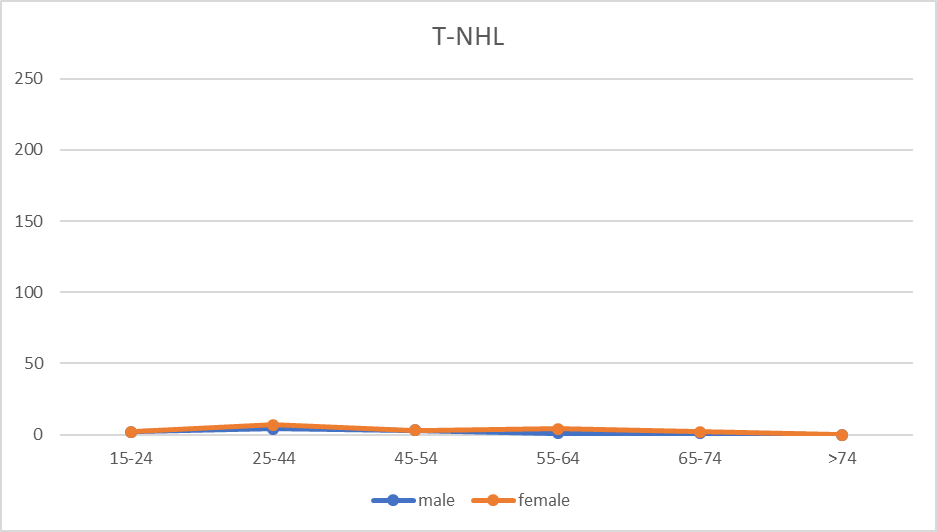

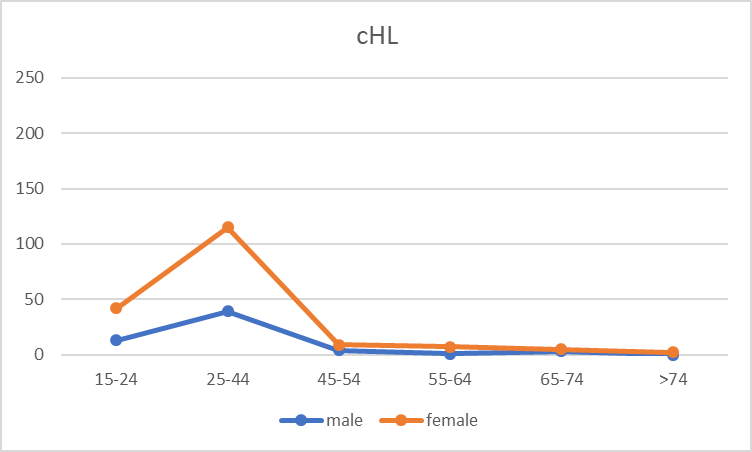
**

**
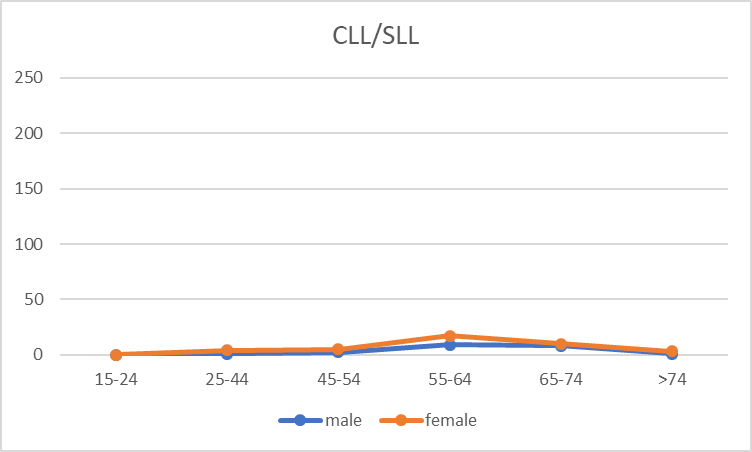

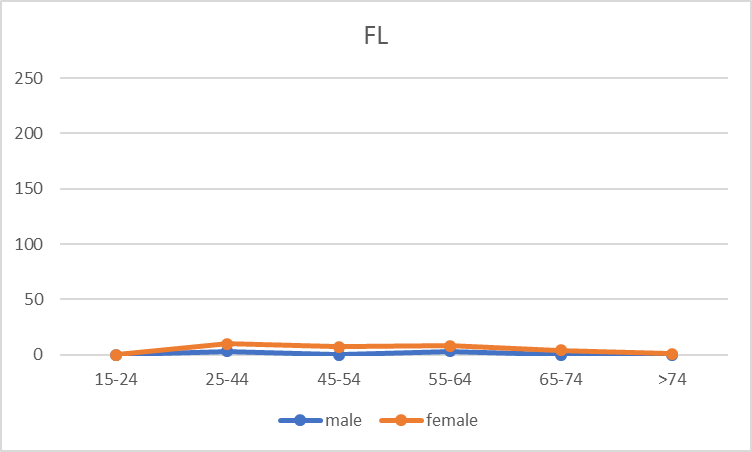
**

**
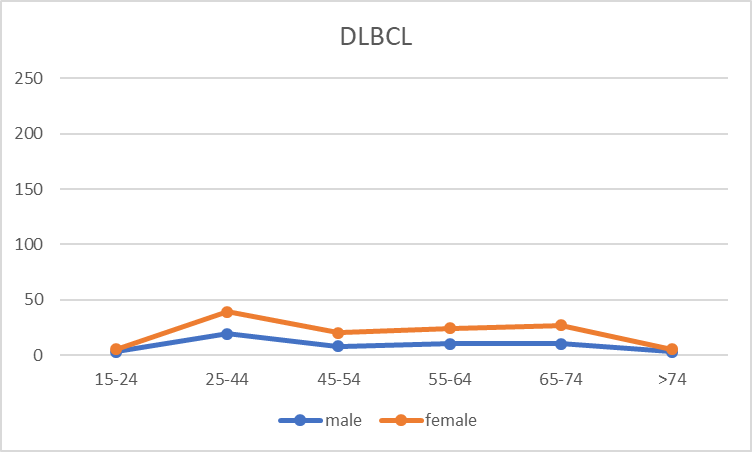

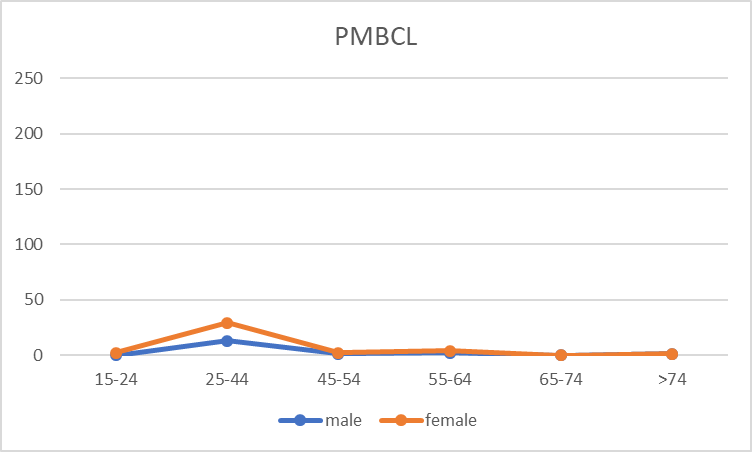
**

**
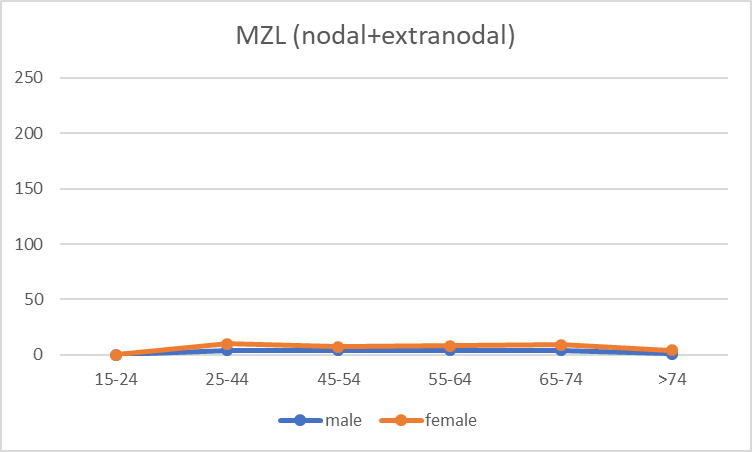

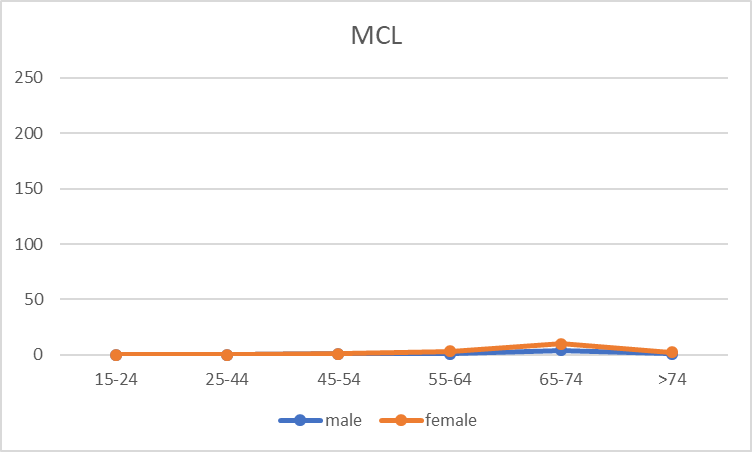
**

Abbreviations. B-NHL: B-cell non-Hodgkin lymphoma; T-NHL: T-cell non-Hodgkin lymphoma; cHL: classical Hodgkin lymphoma; CLL/SLL: Chronic lymphocytic leukemia/small lymphocytic lymphoma; FL: follicular lymphoma; DLBCL: diffuse large B-cell lymphoma; PMBCL: primary mediastinal B-cell lymphoma; MZL: marginal zone lymphoma; MCL: mantle cell lymphoma.
